# Targeting dysregulated lipid metabolism in the tumor microenvironment

**DOI:** 10.1007/s12272-023-01473-y

**Published:** 2023-12-07

**Authors:** Do-Hee Kim, Na-Young Song, Hyungshin Yim

**Affiliations:** 1 Department of Chemistry, College of Convergence and Integrated Science, Kyonggi University, Suwon, 16227 Korea; 2 Department of Applied Life Science, The Graduate School, BK21 Four Project, Yonsei University, Seoul, 03722 Korea; 3 Department of Oral Biology, Yonsei University College of Dentistry, Seoul, 03722 Korea; 4 Department of Pharmacy, College of Pharmacy, Institute of Pharmaceutical Science and Technology, Hanyang University, Ansan, 15588 Korea

**Keywords:** Tumor microenvironment, Lipid uptake, Lipolysis, Lipogenesis, Cholesterol transport, Phytochemical

## Abstract

The reprogramming of lipid metabolism and its association with oncogenic signaling pathways within the tumor microenvironment (TME) have emerged as significant hallmarks of cancer. Lipid metabolism is defined as a complex set of molecular processes including lipid uptake, synthesis, transport, and degradation. The dysregulation of lipid metabolism is affected by enzymes and signaling molecules directly or indirectly involved in the lipid metabolic process. Regulation of lipid metabolizing enzymes has been shown to modulate cancer development and to avoid resistance to anticancer drugs in tumors and the TME. Because of this, understanding the metabolic reprogramming associated with oncogenic progression is important to develop strategies for cancer treatment. Recent advances provide insight into fundamental mechanisms and the connections between altered lipid metabolism and tumorigenesis. In this review, we explore alterations to lipid metabolism and the pivotal factors driving lipid metabolic reprogramming, which exacerbate cancer progression. We also shed light on the latest insights and current therapeutic approaches based on small molecular inhibitors and phytochemicals targeting lipid metabolism for cancer treatment. Further investigations are worthwhile to fully understand the underlying mechanisms and the correlation between altered lipid metabolism and carcinogenesis.

## Introduction

The tumor microenvironment (TME) is a highly heterogeneous environment consisting of many distinct cell types and molecules released by tumor cells, cancer-associated fibroblasts (CAFs), and infiltrating immune cells. The representative cells that surround and support cancer cells in the TME include CAFs, tumor-endothelial cells (TECs), cancer-associated adipocytes (CAAs), tumor-associated neutrophils (TANs), and tumor-associated macrophages (TAMs) (Chen et al. 2015). In addition, non-cancer cells such as endothelial cells, adipocytes, immune/inflammatory cells, and myeloid-derived suppressor cells (MDSCs) can interact directly and/or indirectly with cancer cells in the TME. This collaborative interplay provides favorable conditions for tumor development, growth, and progression (Gonzalez et al. 2018; Petrova et al. 2018; Cao 2019; Cheng et al. 2019; Greten and Grivennikov 2019; Bui et al. 2021). Indeed, numerous studies have demonstrated that the communication between cancer cells and surrounding cells in the TME promotes cancer metastasis and confers chemoresistance through the establishment of metabolic reprogramming, which involves the alteration of tumor metabolism (DeBerardinis and Chandel 2016; Chen et al. 2022; Li et al. 2022; Liu et al. 2022a, b). Metabolic reprogramming and alteration of oncogenic signaling pathways related to metabolic processes have emerged as hallmarks of cancer (Zhu et al. 2022). Thus, it would be essential to understand the metabolic reprogramming related to oncogenic progression to develop strategies for cancer treatment. In this review, we focus on the critical factors in the TME driving lipid metabolic reprogramming to encourage the progression of cancer. In addition, we explore the latest insights and current therapeutic approaches based on small molecular inhibitors and phytochemicals targeting lipid metabolism for cancer treatment because of few reviews summarizing cancer lipid metabolism by phytochemicals.

## Altered lipid metabolism in cancer cells of the TME

Tumor cells need to rewire their metabolic pathways to regulate nutrient intake and metabolism to maintain energy production. Lipids are not only essential energy sources but also membrane components necessary for tumor growth and metabolism. In addition, lipids mediate cell signaling pathways and control redox homeostasis. Numerous proteins and genes responsible for lipid metabolism are regulatory mediators of tumor survival and progression, as well as mutual communication between cancer cells and the TME (Ventura et al. 2015; Gupta et al. 2017). Lipid metabolism is defined as a complex set of molecular processes, including lipid uptake, synthesis, transport, and degradation (Harayama and Riezman 2018). The dysregulation of lipid metabolism is affected by enzymes and signaling molecules that are directly or indirectly involved in the lipid metabolic process. Such imbalance can alter membrane composition, gene expression, and downstream signaling pathway activity, which control cell proliferation, motility, inflammation, and survival (Zechner et al. 2012; Harayama and Riezman 2018). Thus, regulation of lipid-metabolizing enzymes has been shown to modulate cancer development and to avoid resistance to anticancer drugs in tumors and the TME (Ventura et al. 2015; Gupta et al. 2017). Exogenous lipids produced by the TME can also affect tumor malignancy and inflammation (Gupta et al. 2017). The following sections describe diverse signaling pathways responsible for lipid metabolic reprogramming-mediated cancer progression, focusing on the related regulatory proteins and genes.

### Lipid uptake by CD36 and its alteration in cancer

Tumor cells require a variety of transporters to mediate the trafficking of lipids for oxidation or to activate oncogenic signaling pathways. Cancer cells tend to uptake fatty acids from the extracellular environment to meet their needs for growth (Zhang et al. 2022a, b). Cluster of differentiation 36 (CD36) as a scavenger receptor is a macrophage receptor for oxidized low-density lipoprotein (Endemann et al. 1993). CD36, also known as fatty acid translocase protein, functions in the binding or trans-membrane uptake of long-chain fatty acids (Abumrad et al. 1993). CD36 is a membrane glycoprotein that consists of two short intracellular domains, two transmembrane segments, and a large extracellular domain with a hydrophobic sequence where lipid ligands bind (Glatz et al. 2010; Pepino et al. 2014; Glatz and Luiken 2018). High expression of CD36 has been observed in diverse cancer types, including liver cancer (Zhao et al. 2018), breast cancer (Yang et al. 2020), colorectal cancer (Drury et al. 2020), and gastric cancer (Jiang et al. 2019). Gyamfi et al. recently reported that CD36 is a key player in the interaction between adipocytes and breast cancer cells, suggesting its potential as a therapeutic target in the TME. Human breast cancer cells co-cultured with adipocytes show upregulation of CD36 expression, with fatty acid import into the cytosol or mitochondria (Gyamfi et al. 2021). Increased CD36 expression activates the STAT3 signaling pathway mediating adipocyte-induced epithelial–mesenchymal transition (EMT) and stemness. In addition, increased CD36 expression occurs with increased fatty acid-binding protein 4 (FABP4) expression, and their direct interaction regulates the import, transport, and metabolism of fatty acid (Gyamfi et al. 2021). Inhibition of CD36 and FABP4 significantly reduces the proliferation, migration, invasiveness, and tumorsphere-forming capacity of breast cancer cells, which is associated with reduced tumorigenicity in a xenograft mouse model (Gyamfi et al. 2021).

The stability and function of CD36 are regulated by post-translational modifications, including palmitoylation, glycosylation, phosphorylation, and ubiquitination (Glatz et al. 2010; Pepino et al. 2014; Glatz and Luiken 2018). Intracellular lipid accumulation by dysregulated lipid metabolism is associated with non-alcoholic steatohepatitis (NASH), a subset of non-alcoholic fatty liver disease (NAFLD) (Chavez-Tapia et al. 2012). Expression of soluble CD36 is significantly increased in patients with advanced steatosis of NAFLD (Garcia-Monzon et al. 2014). Palmitoylation is a covalent attachment of palmitate to cysteine residues of the protein, and increased palmitoylation of CD36 in NASH facilitates fatty acid uptake and lipid accumulation (Zhao et al. 2018). Inhibition of CD36 palmitoylation interferes with the binding and uptake of fatty acids. It alleviates liver tissue inflammation by inactivating the JNK signaling pathway while interfering with CD36/Fyn/Lyn complex formation (Zhao et al. 2018). In addition, *O*-linked β-*N*-acetylglucosamine (*O*-GlcNAc) glycosylation called an *O*-GlcNAcylation is modulated by *O*-GlcNAc transferase (OGT) and *O*-GlcNAcase (OGA), which are responsible for *O*-GlcNAc addition and removal (Zachara and Hart 2002). *O*-GlcNAcylation modulates protein functions by regulating subcellular localization, protein stability, transcriptional activity, and protein–protein interaction (Zachara and Hart 2002; Chatham et al. 2021). Dysregulation in *O*-GlcNAc cycling has been implicated in the progression of various chronic human diseases related to inflammation and tumor growth (Chatham et al. 2021; Ouyang et al. 2022). Interestingly, elevated fatty acid uptake activity promotes the metastatic potential of gastric cancer cells in a CD36-dependent manner via upregulation of *O*-GlcNAcylation (Jiang et al. 2019). Increased *O*-GlcNAcylation level accelerates the transcription of CD36 by activating the NF-κB pathway, and CD36 can be directly modified at Ser468 and Thr470 residues by *O*-GlcNAc (Jiang et al. 2019). Consequently, the induction of CD36 expression facilitates fatty acid uptake in gastric cancer cells, forming a vicious cycle between *O*-GlcNAcylation and CD36 transcription. This relationship may be correlated with poor survival in gastric cancer (Jiang et al. 2019).

Recently, the role of CD36 has been receiving attention not only in cancer cells but also in immune cells within TME. Intratumoral regulatory T (Treg) cells exhibit expression of metabolic genes associated with lipid metabolism compared to circulating Treg cells. Intratumoral Treg cells, in particular, show a substantial increase in the expression of CD36 (Wang et al. 2020a, b, c). CD36-mediated metabolic adaptation supports the survival and suppressive functions of intratumoral Treg cells, which is mediated by mitochondrial fitness and NAD production (Wang et al. 2020a, b, c). In contrast, it has been demonstrated that CD8+ tumor-infiltrating lymphocytes were shown to have reduced anti-tumor function, which is attributed to an increase in lipid peroxidation resulting from the uptake of fatty acids and oxidized lipids mediated by CD36 (Ma et al. 2021; Xu et al. 2021). In addition, tumor-associated macrophages (TAMs) residing in TME accumulate lipids through enhanced lipid uptake via CD36. This accumulation leads to the differentiation and functional alteration of pro-tumoral M2 type TAMs characterized by high levels of fatty acid oxidation (Su et al. 2020a, b; Yang et al. 2022). Therefore, further research and understanding are needed on the regulatory mechanism involving post-translational modifications of CD36 in controlling cell–cell communication within the TME since CD36 is a potential biomarker and therapeutic target for cancer.

### Alteration of lipogenesis in cancer

Tumor cells obtain fatty acids from de novo lipogenesis and require activation of fatty acid synthesis to meet biosynthetic and bioenergetics requirements during carcinogenesis and tumorigenesis (Mounier et al. 2014). Lipogenesis encompasses the process of fatty acid triglyceride synthesis from acetyl-CoA synthesized by glucose or other substrates. Acetyl-CoA is carboxylated by acetyl-CoA carboxylase 1 (ACC1), converting to malonyl-CoA. ACC1 is located in the cytoplasm and acts as a rate-limiting enzyme in de novo fatty acid synthesis, producing malonyl-CoA as a substrate for forming palmitate catalyzed by fatty acid synthase (FASN) (Wakil and Abu-Elheiga 2009). ACC2 localizes in the mitochondria and regulates the activity of carnitine palmitoyltransferase 1 (CPT1), involved in the β-oxidation of fatty acids (Wakil and Abu-Elheiga 2009). Malonyl-CoA acts as an inhibitor of the CPT1, offering a pathway choice between fatty acid synthesis and β-oxidation. In addition, stearoyl-CoA desaturase 1 (SCD1) is a Δ^9^ desaturase, which catalyzes the insertion of a *cis* double bond at the Δ^9^ position of 12–19 carbon saturated fatty acids, converting them to monounsaturated fatty acids. SCD1 affects lipid composition, membrane fluidity, and functionality, which are essential for maintaining cellular integrity (Rodriguez-Cuenca et al. 2016). Sterol regulatory-element binding proteins (SREBPs) are membrane-bound transcription factors that induce the expression of genes related to lipogenesis and cholesterol biosynthesis (Schiliro and Firestein 2021). The expression of enzymes and genes involved in lipogenesis is associated with oncogenic signaling in tumors, facilitating immune system evasion, tumor growth, and chemoresistance (Flaveny et al. 2015).

#### Acetyl-CoA carboxylase 1 (ACC1)

ACC1 is abundantly expressed in various cancer cells, and its overexpression is associated with poor prognosis in cancer patients. ACC1 knockdown using siRNA inhibits the proliferation of highly lipogenic prostate cancer LNCaP, glioblastoma U87 EGFRvIII cell lines, colon cancer HCT-116, and liver cancer HepG2 cells (Brusselmans et al. 2005; Zhan et al. 2008; Jones et al. 2017; Ye et al. 2019a, b) but does not affect the proliferation of normal cells (Brusselmans et al. 2005). In addition, the expression level of ACC1 positively correlates with prolyl isomerase Pin1 expression in human prostate cancer specimens (Ueda et al. 2019). Pin1 recognizes the phosphorylated Ser/Thr-Pro motif and facilitates the cis–trans isomerization of proline (Stukenberg and Kirschner 2001). Direct binding of ACC1 to the WW domain of Pin1 improves the stability and activity of ACC1 protein by blocking lysosome-mediated degradation (Ueda et al. 2019). High expression of ACC1 protein in prostate cancer cells is caused by Pin1-mediated posttranscriptional level, contributing to increased FA contents that support cell proliferation (Ueda et al. 2019). Moreover, ACCs reconstruct lipogenesis-dependent cancer metabolism in head and neck squamous cell carcinoma (HNSCC), so HNSCC cells can survive inhibition of the Warburg effects by cetuximab treatment (Luo et al. 2017). HNSCC cells with acquired cetuximab resistance exhibit increased expression and activation of ACC1. Combination treatment with ACC allosteric inhibitor TOFA and cetuximab produces a more potent anti-tumor effect of cetuximab-resistant HNSCC xenograft (Luo et al. 2017).

Tumor-specific CD4+ T cells can support therapeutic effects by maintaining effector CD8+ T cells, which are important to reduce the exhaustion of CD8+ T cells mediated by PD-1 and TRAIL-mediated apoptosis during initial tumor elimination (Church et al. 2014). It has been reported that differentiation of Th17 cells depends on ACC1-mediated de novo fatty acid synthesis (Berod et al. 2014; Endo et al. 2015). The function of Th17 cells in TME remains unclear, but it can favor tumor growth by promoting angiogenesis or suppressing tumor immunity. Inhibition of fatty acid biosynthesis by T cell-specific deletion of ACC1 in mice attenuates Th17 cell-mediated inflammatory diseases (Berod et al. 2014). Furthermore, genetic deletion of ACC1 enhances the formation of memory CD4+ T cells, which exhibits the phenotype characterized by low fatty acid biosynthesis and high respiratory capacity (Endo et al. 2019).

Although ACC1 is recognized as an oncoprotein in various cancer cells including prostate cancer, glioblastoma, colon cancer, and liver cancer (Brusselmans et al. 2005; Zhan et al. 2008; Jones et al. 2017; Ueda et al. 2019; Ye et al. 2019a, b), there is evidence that ACC1 can act as a tumor suppressor by modulating the energy homeostasis or attenuating protein acetylation in tumor xenograft models (Jeon et al. 2012; Rios Garcia et al. 2017). Under metabolic energy stress conditions, AMPK can accelerate the survival of lung cancer by governing the NADPH level and maintaining the ATP level. Knockdown of ACC1 reduces the NADP^+^/NADPH ratio and ROS level, which promotes tumor formation in the lung cancer xenograft model using human A549 cells (Jeon et al. 2012). In addition, ACC1 inactivation by leptin or TGFβ treatment drives the invasion and metastasis of breast cancer cells, which is mediated by the elevation of acetyl-CoA production. Subsequently, protein acetylation, particularly transcription factor Smad2, leads to the upregulation of EMT markers such as vimentin and N-cadherin (Rios Garcia et al. 2017). In acute myeloid leukemia (AML), the Trib1-COP1 complex, an E3 ubiquitin ligase, is a substrate for ACC and induces proteasome-mediated degradation. Its degradation may initiate metabolic reprogramming to support the energy source for the progression of leukemia cells (Ito et al. 2021). Moreover, the stabilization of ACC1 suppresses the growth of human AML cells by inducing loss of self-renewal activity in leukemia-initiating cells, which is mediated by increased ROS levels and NADPH consumption (Ito et al. 2021). Based on previous reports, further detailed studies are needed to understand the physiology of ACC1, which can act as either a tumor suppressor or an oncogene depending on the complexity of the TME in terms of energy balance.

#### Fatty acid synthase (FASN)

High expression of FASN, a critical regulator of lipid metabolism, is associated with cancer progression in various types of cancer in the breast, prostate, ovary, and liver (Flavin et al. 2010; Fhu and Ali 2020). FASN acts as an oncogenic factor due to its role in regulating fatty acid synthesis or inducing aberrant lipogenesis in cancer cells (Che et al. 2020). FASN has been identified as a poor prognostic factor in patients with colon cancer, cervical cancer, cholangiocarcinoma, and clear cell renal cell carcinoma (Yuan et al. 2020; Tomacha et al. 2021; Drury et al. 2022; Du et al. 2022). FASN catalyzes the biosynthesis of the fatty acid palmitate, which accelerates RhoU palmitoylation, a critical step in regulating the turnover of focal adhesions (Baenke et al. 2013). Silencing FASN in prostate cancer leads to a reduction of RhoU palmitoylation and decreases the protein stability of Cdc42, which is connected to the suppression of migration and invasion (De Piano et al. 2020, 2021). In cholangiocarcinoma, the knockdown of FASN suppresses cell growth, migration, and invasion, which is associated with decreased cellular levels of palmitic amide, a fatty acid amide derived from palmitic acid (Tomacha et al. 2021). In addition, FASN promotes lymph node metastasis via cholesterol reprogramming and lymphangiogenesis in cervical cancer. FASN activates the c-Src-PI3K-Akt-FAK signaling pathway via cholesterol reprogramming, defined as lipid rafts and actin skeleton remodeling, leading to enhanced cell migration and invasion (Du et al. 2022). FASN also induces lymphangiogenesis by secreting PDGF-AA and IGFBP-3 (Du et al. 2022). Moreover, mutant K-Ras has been shown to stimulate lipogenesis by controlling lipogenic enzymes, including ACC1 and FASN (Kerk et al. 2021). Knockdown of K-Ras suppresses the levels of ACC1 and FASN expression, contributing to the suppression of spheroid formation through ROS production in K-Ras-activated pancreatic cancer cells (Terado et al. 2022).

Recently, Schroeder et al*.* reported that the network between fatty acid biogenesis catalyzed by FASN and the interactions within the Bcl-2 family controls the mitochondrial priming response to apoptosis (Schroeder et al. 2021). Inhibition of FASN promotes an increase in the NADPH/NADP^+^ ratio in breast cancer cells; the redox imbalance leads to the activation of stress-related proapoptotic kinases such as JNK and p38 MAPK as well as energy-sensing AMP-activated protein kinase (AMPK) (Schroeder et al. 2021). Interestingly, pharmacological inhibition of FASN activity results in an upregulation of mRNA and protein levels of the BH3-only Bcl-2 members such as Bim, NOXA, and PUMA (Schroeder et al. 2021). These findings suggest that FASN inhibitory cancer cells acquire increased mitochondrial apoptotic priming that can induce apoptotic hypersensitivity to Bcl-2-specific BH3-mimetics.

Post-translational modifications such as ubiquitination and sumoylation of FASN protein protect FASN from proteasomal degradation (Fhu and Ali 2020). Ubiquitin-specific proteases (USPs) are responsible for cellular functions through interactions with various target proteins, and their expressions are correlated with worse prognosis in several human cancers (Cruz et al. 2021). FASN can interact with USPs and prevent ubiquitin-mediated degradation among several target proteins. Androgen-stimulated USP2a upregulation in prostate cancer has been shown to stabilize FASN expression by blocking its polyubiquitination (Graner et al. 2004). Another study demonstrated that interaction between USP38 and FASN enhances the stability of FASN protein and increases triglyceride production in gastric cancer cells, which contributes to cell proliferation, migration, and tumorigenesis (Zheng et al. 2022).

Elevated FASN expression in ovarian cancer cells results in the accumulation of lipids and subsequent inhibition of tumor-infiltrating dendritic cells, which are required to sustain the capacity of anti-tumor T cells (Jiang et al. 2018). Hence, by targeting FASN, anti-tumor immunity can be enhanced by reducing lipid accumulation-induced dysfunction in dendritic cells (Jiang et al. 2018). In addition, intratumoral Treg cells display elevated SREBP activity, which results in FASN-dependent fatty acid synthesis (Lim et al. 2021). Deletion of FASN in Treg cells inhibits the growth of MC38 and B16 tumors (Lim et al. 2021). Interestingly, the heightened expression of PD-1 in Treg cells infiltrating tumors depends on SREBP activation, indicating a potential avenue for cancer immunotherapy targeting the lipid metabolism of Treg cells (Lim et al. 2021). Recent reports indicate that activated fatty acid metabolism by FASN is associated with reduced immune infiltration in male breast cancer, leading to the promotion of metastasis (Sun et al. 2023). These studies support FASN as a critical factor in abnormal lipid metabolism of cancer progression, and further detailed studies are needed to understand the function of FASN in reprogramming lipid metabolism.

#### ATP-citrate lyase (ACLY)

ACLY is a nuclear-cytosolic homotetrameric enzyme that catalyzes the production of cytosolic acetyl-CoA, generating a substrate for de novo biosynthesis of fatty acids and cholesterol (Chypre et al. 2012). Acetyl-CoA provides an acetyl donor for the acetylation of cytosolic and nuclear proteins, including transcription factors and histones, which control gene expression related to cancer metabolism (Wellen et al. 2009). ACLY is highly expressed or activated in several cancers, supporting tumor cell growth through lipogenesis (Bauer et al. 2005; Zaidi et al. 2012). ACLY depletion in various cancer cell lines exerts an anti-proliferative effect through the generation of mitochondrial ROS and AMPK activation accompanied by triglyceride accumulation and down-regulation of carnitine palmitoyltransferase 1A (Migita et al. 2013, 2014). In addition, glucose injection induces acetylation of lysine residues of ACLY in lung cancer A549 cells and mouse liver, blocking ubiquitination and increasing ACLY protein expression (Lin et al. 2013). Lung cancer tissues consistently exhibit enhanced ACLY acetylation that contributes to lipogenesis and promotes tumor cell proliferation (Lin et al. 2013).

As mentioned earlier, ACLY is a nucleocytosolic enzyme that produces acetyl-CoA from citrate and plays a critical role in determining the level of histone acetylation. ACLY phosphorylation activated by Akt promotes histone acetylation in both cancer and immune cells present in TME, inducing their proliferation (Lee et al. 2014; Covarrubias et al. 2016; Osinalde et al. 2016). Histone acetylation in human glioma and prostate tumors responds to acetyl-CoA production levels in the nucleus, which is accelerated upon increased phosphorylation of ACLY by Akt (Lee et al. 2014). In addition, M2 macrophage activation can be induced by IL-4-triggered acetyl-CoA production and histone acetylation as a consequence of Akt-mediated ACLY activation (Covarrubias et al. 2016). Recently, it has been increasingly emphasized that intracellular acetyl-CoA level in TME plays a vital role in regulating the fate of T cells (Vodnala et al. 2019). Phosphorylation of ACLY is induced upon IL-2-triggered Akt activation and generates acetyl-CoA that serves as a substrate for histone acetyltransferases. It stimulates the acetylation of histones in promoters of cell cycle-related genes, causing T-cell proliferation (Osinalde et al. 2016). IL-12-stimulated CD8^+^ T cells exhibit increased intracellular acetyl-CoA levels due to high expression of ACLY, resulting in the maintenance of IFNγ production and lipid biosynthesis for energy demand in nutrient-deprived TME (Chowdhury et al. 2022).

#### Acyl-CoA synthetase short-chain family member 2 (ACSS2)

ACSS2, one of the acyl-CoA synthetase short-chain family members, is a nucleocytosolic enzyme that catalyzes the conversion of acetate to acetyl-CoA, which is highly expressed in various tumors (Liu et al. 2022a, b). It has been proven through in vitro and in vivo experiments that ACSS2 plays an important role in tumor cell survival under hypoxia in lung cancer, breast cancer, melanoma, and colon cancer (Yoshii et al. 2009). ACSS2 is a critical enzyme that supplies a key source of acetyl-CoA for tumors using acetate as a carbon source to maintain lipid synthesis and histone acetylation (Comerford et al. 2014; Bulusu et al. 2017). SREBP transcriptionally upregulates ACSS, and this upregulation is highly expressed in tumor cells, as a response to the acidic TME (Kondo et al. 2017). In addition, under metabolic stress such as hypoxia or low serum, ACSS2 is required for the utilization and uptake of acetate and also supports the biosynthesis of membrane phospholipids for tumor cell growth (Kamphorst et al. 2014; Schug et al. 2015; Gao et al. 2016). Silencing *ACSS2* caused significant inhibition of spheroid formation and markedly reduced xenograft tumor growth derived from breast and prostate cancer cells (Schug et al. 2015). In addition, ACSS2 induced histone acetylation at the *FASN* promoter region, which upregulated FASN expression to enhance lipid synthesis to promote the survival of hepatocellular carcinoma (Gao et al. 2016). ACSS2 is also overexpressed in malignant plasma cells derived from patients with myeloma. In particular, the expression of ACSS2 is significantly higher in obese myeloma patients (Li et al. 2021). Treatment of adipocyte-secreted angiotensin II enhanced the expression of ACSS2 in myeloma cells, which promotes tumorigenesis by maintaining the stability of interferon regulatory factor 4 (IRF4), an oncogenic protein (Li et al. 2021). ACSS2-mediated IRF4 acetylation results in the elevation of IRF4 protein level due to dysregulation of p62-mediated lysosomal degradation in myeloma (Li et al. 2021). Moreover, the acetylation of HIF-2α in the presence of ACSS2 significantly increased the expression level of HIF-2α target genes, potentially linking tumor cell growth and metastasis (Chen et al. 2017; Nagati et al. 2019).

Like ACLY, enforced expression of ACSS2 in T cells increased acetate-derived carbon incorporation in citrate and fatty acid, whereas reducing ACSS expression in T cells impairs IFN-γ production by tumor-infiltrating lymphocytes and tumor clearance (Qiu et al. 2019). Acetate promotes histone acetylation and restores chromatin accessibility in glucose-limited T cells, following enhanced IFN-γ production in an ACSS-dependent manner (Qiu et al. 2019).

#### Stearoyl-CoA desaturase 1 (SCD1)

Stearoyl-CoA desaturase-1 (SCD1) converts saturated fatty acids (SFA) into monounsaturated fatty acids (MUFA), which are used for the synthesis of phospholipids, triglycerides, and cholesterol esters, important components of membrane phospholipids. The MUFA/SFA ratio influences the plasma membrane fluidity and signal transduction (Kim and Ntambi 1999). Knockdown of SCD1 in human lung adenocarcinoma A549 cells reduced the MUFA/SFA ratio in cell membrane lipids, leading to the inactivation of Akt signaling, impairing lipogenesis (Scaglia and Igal 2008). Mice inoculated with SCD1-depleted A549 cells showed a reduction in tumor development compared with control cell-injected tumors (Scaglia and Igal 2008). In addition, inhibition of SCD1 activity suppressed migration and invasion by regulating EMT in colorectal cancer HCT116 cells harboring high expression levels of SCD1, which might be related to reduced level of MUFA and the MUFA/SFA ratio in SCD1 knockdown cancer cells (Ran et al. 2018). SCD1 is highly expressed in a variety of human cancer tissues including breast, prostate, lung, colon, kidney, and ovarian cancer (Roongta et al. 2011; von Roemeling et al. 2013; Ran et al. 2018), and reports have shown SCD1 overexpression to function as an oncogene in various cancers and to predict poor clinical outcome (Huang et al. 2016; Wang et al. 2016; Wang et al. 2020a, b, c).

Acidosis of TMEs contributes to tumor progression, invasion, and resistance to chemotherapy (Kato et al. 2013). Interestingly, melanoma (Mel501) cells exhibited a higher level of unsaturated species than saturated fatty acids under acidic conditions, which appears to be associated with the upregulation of SCD expression (Urbanelli et al. 2020). Acidification activates the PI3K/Akt signaling pathway to increase the expression of SCD1, which then binds to PPARα and promotes hepatic tumorigenesis through lipid metabolic reprogramming (Ding et al. 2022). In addition, the treatment of cobalt chloride, a hypoxia imitative agent, induced the expression of *SCD-1* mRNA in human clear cell renal carcinoma Caki-2 cells, accompanied by increased mRNA levels of *HIF1A* and *HIF2A* (Melana et al. 2021). Moreover, the conversion of SFA (stearic acid; 18:0) into MUFA (oleic acid; 18:1n-9) is promoted under hypoxic conditions, which favors tumor cell proliferation (Melana et al. 2021). Furthermore, there is clinical significance of SCD1 and FABP4 expression in primary human tumors and metastatic tissues that relapse after first-line chemo- or hormone therapy in breast cancer patients. Notably, FABP4 was detected in blood vessels and adipocytes adjacent to metastatic relapse tissue of human breast cancer patients (Luis et al. 2021). In the syngeneic Lewis lung carcinoma (LLC) mouse tumor model and mouse xenograft model using MDA-MB-231 breast cancer cells, FABP4 expression was upregulated by hypoxia and re-oxygenation in adipocytes and tumor endothelial cells but not in cancer cells (Luis et al. 2021). Conversely, SCD1 expression was high in tumor sections, which leads to fatty acid desaturation, supporting cancer cell membrane fluidity and migration (Luis et al. 2021). FABP4-induced lipid droplet formation in cancer cells has been shown to provide a resource for survival under hypoxia and oxidative stress-induced ferroptosis, which might promote tumor recurrence (Luis et al. 2021).

In contrast to the previous reports, Ducheix et al*.* reported that intestinal-specific *Scd1* (i*Scd1*) knockout mice are prone to trigger inflammation and cancer in the gut compared with controls, in response to decreased hepatic MUFA proportion (Ducheix et al. 2018, 2022). In a diethylnitrosamine-induced model of HCC, disturbance of the hepatic fatty acid profile induced by i*Scd1* deletion favors tumor progression of extensive hepatic tumors and more frequent metastasis in the lungs compared with wild-type mice (Ducheix et al. 2022). Depending on the type of cancer, the physiology of SCD1 can differ. Further investigations into the functions of SCD1 in the lipid metabolism of individual cancer would contribute to the development of anticancer drugs.

### Lipolysis and cancer progression

#### ATGL and HSL

Lipolysis is the metabolic process of hydrolyzing triglyceride (TG) into glycerol and free fatty acids. Several factors are related to lipolysis and cancer. Adipose triglyceride lipase (ATGL) and hormone-sensitive lipase (HSL) are key enzymes involved in the breakdown of intracellular TG, providing free fatty acids that can provide energy for cancer cell growth and migration (Zechner et al. 2012). ATGL is found in breast cancer cells, and its higher expression level is associated with more aggressive tumors (Wang et al. 2017). Its expression is amplified upon interaction with adipocytes to trigger the release of free fatty acids that are channeled into fatty acid β-oxidation. This metabolic process is prevalent in cancer cells but not in normal breast epithelial cells (Wang et al. 2017). In addition, increased expression of ATGL accelerates the growth of CRC cells, while its suppression enhances apoptosis in these cells. ATGL promotes the lipolytic process in CRC cells. Overexpression of ATGL in SW480 cells leads to increased levels of FFA and decreased levels of TAG compared to control cells. Conversely, HCT116 cells with ATGL knockdown exhibit decreased levels of FFA. ATGL-mediated lipolysis provides FFA for cholesterol metabolism and CoA biosynthesis (Yin et al. 2021). ATGL level is elevated in human colonic tumors, and their expression is further amplified by obesity. The increase in ATGL, influenced by the obesity-related compound oleic acid, enhances cancer cell migration. This migratory effect can be mitigated by inhibiting ATGL, which contributes to broad transcriptional changes in human colon cancer cells related to growth and metabolism (Iftikhar et al. 2021). However, the genetic ablation of neutrophil *ATGL* in the orthotopic 4T1 tumor model results in an increase in lung metastases of breast cancer cells (Li et al. 2020). The authors suggest that it would be intriguing to elucidate the roles played by bioactive lipid mediators originating from lung neutrophils within the lung metastatic niche, as well as to investigate the potential transfer of lipids from neutrophils to tumor cells in TME (Li et al. 2020).

The level of HSL mRNA in adipose tissue of cancer patients showed a notable increase compared to control patients. Additionally, cancer patients displayed a twofold elevation in both serum triacylglycerol and serum free fatty acid levels (Thompson et al. 1993). However, a study by Xu and colleagues recently revealed that HSL deficiency correlates with inflammation in both adipose tissue and the pancreas, accelerating pancreatic ductal adenocarcinoma (PDAC) in the Kras^G12D^ mouse model (Xu et al. 2018). Administration of HSL inhibitors has been proposed for the management of cachexia (Das et al. 2011). Additionally, the precise effect of chronic HSL suppression on the progression of PDAC needs to be investigated.

#### FABP4

FABP4, known as adipocyte fatty acid binding protein, exhibits high expression in adipocytes. During the adipocyte differentiation process, FABP4 interacts with HSL to regulate lipolysis (Furuhashi 2019). Exogenous FABP4 interacts with adipocytes, promoting differentiation and facilitating p38/HSL-mediated lipolysis (Dou et al. 2020). Additionally, FABP4 plays a significant role in tumor transformation, proliferation, metastasis, and drug resistance by enhancing lipid transport (Guaita-Esteruelas et al. 2018). Co-culture of cancer cells with adipocytes triggers lipolysis in adipocytes, leading to subsequent β-oxidation through lipid transfer to cancer cells, which has been demonstrated in breast and ovarian cancers (Nieman et al. 2011; Attane et al. 2020). FABP4 can bind reversibly to long-chain fatty acids and is highly expressed in adipocytes (Furuhashi and Hotamisligil 2008). FABP4 expression is strongly exhibited at the interface between adipocyte and ovarian cancer cells, enhancing lipid availability in cancer cells and promoting rapid tumor growth and metastasis (Nieman et al. 2011). In a co-culture model involving ovarian cancer cells and adipocytes, treatment with an FABP4 inhibitor attenuated lipid accumulation in the cancer cells, reducing migration and invasion of cancer cells facilitated by adipocytes (Nieman et al. 2011). A link between FABP4 and lipolysis in the survival and growth of cancer cells through adipocytes has also been reported in breast cancer tissues (Kim et al. 2020).

#### MIC-1

Macrophage inhibitory cytokine-1 (MIC-1), a stress response cytokine, belongs to the transforming growth factor beta (TGF-β) superfamily as a divergent member (Bootcov et al. 1997). MIC-1 is recognized as a protumorigenic marker associated with progressive prostate cancers. Studies have consistently demonstrated its presence in prostate cancer tissues, influencing cell proliferation and invasion (Bauskin et al. 2006; Bruzzese et al. 2014; Jones et al. 2015). Notably, the role of a high-fat diet in prostate cancer progression has been studied (Huang et al. 2012, 2014; Wu et al. 2016). A high-fat diet markedly enhanced adipocyte infiltration and adipose lipolysis in a mouse intraperitoneal xenograft model of prostate cancer, which facilitates tumor progression through the expression and secretion of MIC-1 (Huang et al. 2021). Furthermore, the co-culture of prostate cancer PC-3 with surrounding adipocytes stimulates the secretion of MIC-1, while prostate stromal fibroblasts release IL-8, both influenced by the fatty acids secreted through activated lipolysis (Huang et al. 2021). Collectively, these findings underscore the intricate interplay between lipid metabolism, the TME, and the expression and effects of MIC-1 in prostate cancer progression.

#### ADM

**Fig. 1:**
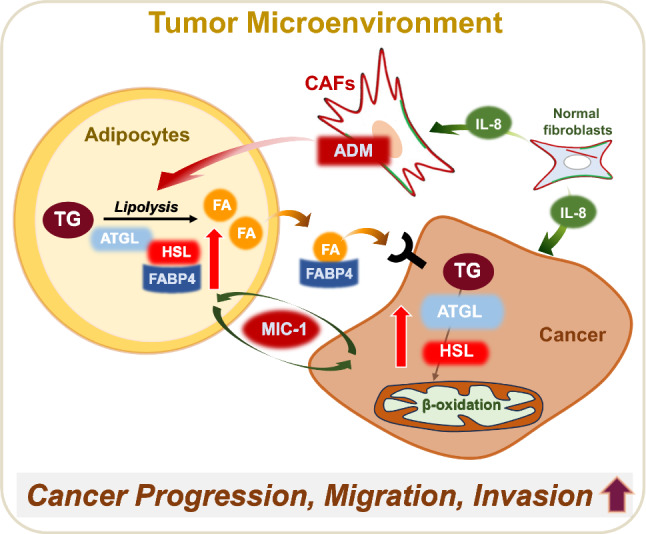
Possible regulatory mechanism of lipolysis in tumor microenvironment. Lipolysis is the metabolic process in which triglyceride (TG) is broken down into glycerol and free fatty acids (FA). Overexpression of ATGL and HSL in adipocyte and cancer cells leads to the release of FAs which are used in fatty acid β-oxidation. Adipocyte fatty acid binding protein (FABP4) can bind to long-chain fatty acids reversibly and triggers lipolysis in adipocytes, enabling lipid transfer to cancer cells and promoting β-oxidation. In addition, the interaction of cancer cells with surrounding adipocytes stimulates the secretion of macrophage inhibitory cytokine-1 (MIC-1) and the release of IL-8 by stromal normal fibroblasts, influenced by the fatty acids secreted through activated lipolysis. Exosomal adrenomedullin (ADM) is a multifactorial, hypoxia-inducible peptide facilitates lipolysis through interaction with adipocytes within the tumor microenvironment. The proteins associated with lipolysis highlight the complex interactions between lipid metabolism and the tumor microenvironment, playing a crucial role in the cancer progression, migration, and invasion

Exosomal adrenomedullin (ADM), a multifactorial hypoxia-inducible peptide derived from pancreatic cancer cells, was shown to induce lipolysis in adipose tissues (Sagar et al. 2016). This effect extends to exosomal ADM derived from CAFs, which promotes lipolysis in both mouse 3T3-L1 and human adipocytes (Kong et al. 2018). Adding to this complex network, recent findings by Pare et al*.*, reveal that MCF-7 mammospheres produce ADM, which modifies the phenotype of cancer-associated adipocytes via the ADM receptor in a paracrine manner. Stimulation of adipocytes by ADM promotes lipolysis through the phosphorylation of hormone-sensitive lipase (HSL) and uncoupling protein 1 (UCP1) expression in breast adipocytes, which may provide energy to cancer cells or remodel the TME (Pare et al. 2020). Therefore, the emerging understanding of the involvement of exosomal ADM in adipose tissue interactions and lipolysis within the TME offers a perspective on the intricate crosstalk among cancer cells, and adipocytes, and their mutual influence. In Fig. 1, we illustrate the potential signaling that may modulate tumor progression through the stimulation of lipolysis in the tumor microenvironment.

### ABC transporter for cholesterol transport and chemoresistance

Biosynthesis of cholesterol begins with acetyl-CoA derived from mitochondria and transported to the cytosol through a series of chemical reactions known as the mevalonate pathway. Condensation of acetyl-CoA with acetoacetyl-CoA forms 3-hydroxy-3-methylglutaryl-CoA (HMG-CoA), which is then converted by HMG-CoA reductase (HMG-CoAR) to mevalonate (Sharpe and Brown 2013) and then further converted to cholesterol (Sharpe and Brown 2013). Cholesterol is an essential constituent of the cellular membrane, which plays an important role in maintaining the stability of the cellular membrane, supporting cell proliferation, and serving as a precursor for the biosynthesis of steroid hormones (Ghayee and Auchus 2007; Hu et al. 2010; Arita et al. 2015). In addition, several studies have suggested that cholesterol accumulation promotes cancer development and resistance to anticancer drugs (Yue et al. 2014; Yan et al. 2020). Cholesterol enriched within the TME leads to increased endoplasmic reticulum stress in CD8+ T cells, resulting in the upregulation of immune checkpoint expression, particularly PD-1, at the transcriptional level. This cholesterol-induced response contributes to the functional exhaustion of CD8+ T cells (Ma et al. 2019). Statin, the inhibitor for cholesterol synthesis, has the capability to transcriptionally suppress PD-L1 expression. As a result, they can mitigate the aggressiveness of NSCLC through ferroptosis (Mao et al. 2022). Moreover, in the meta-analysis of NSCLC immunotherapy cohorts, stains enhance the drug-response effect of anti-PD-1 therapy and prolong the survival of NSCLC patients (Mao et al. 2022).

#### ABCA1

ATP-binding cassette (ABC) transporters, one of the most prominent families of integral plasma membrane proteins, are involved in cholesterol transport across the cell membrane. Notably, the relationship between ABC transporter A1 (ABCA1) and cholesterol is becoming prominent in cancer (Pasello et al. 2020). In response to elevated cellular cholesterol levels, ABCA1 expression is upregulated by blocking the ubiquitination and subsequent proteasomal degradation, leading to an increase in free cholesterol efflux (Hsieh et al. 2014). The expression of ABCA transporters is mainly regulated by the sterol regulatory element-binding protein 2 (SREBP2) and liver X receptor (LXR) (Goldstein et al. 2006; Gabbi et al. 2014). It has been reported that cholesterols are converted to oxysterols and prevent the translocation of SREBP2 to the nucleus, leading to *ABCA1* expression. LXR acts as a sensor of cholesterol homeostasis and interacts with retinoid X receptor (RXR), which forms a heterodimeric complex under high-cholesterol conditions (Costet et al. 2000; Venkateswaran et al. 2000). The implications of ABC transporter expression in cancer are controversial. In prostate cancer cells, the reduced expression of *ABCA1* with promoter hypermethylation maintains elevated intracellular cholesterol levels, contributing to aggressive prostate cancer progression (Lee et al. 2013). However, Gao et al*.* reported that ABCA1 suppression impairs malignant phenotypes of epithelial ovarian cancer (EOC) cells and reduces the formation of EOC spheroids, which may be associated with the induction of intracellular cholesterol (Gao et al. 2022). In triple-negative breast cancer, elevated cholesterol levels from ABCA1 knockdown in membranes of breast cancer cells diminished membrane fluidity, hindering cell migration (Zhao et al. 2016). This process might be associated with apoptosis triggering in cholesterol-rich environments (Gajate and Mollinedo 2001). Cholesterol efflux mediated by ABCA1 overexpression promoted EMT and increased invasive capacity by maintaining caveolin-1 stability in colorectal cancer, the expression level of which could be used as a prognostic biomarker in colon cancer patients (Aguirre-Portoles et al. 2018).

#### ABCB1

ABC transporter B1 (ABCB1), also known as multidrug resistance protein 1 (MDR1) or P-glycoprotein, facilitates cholesterol distribution in the plasma membrane using floppase (Le May et al. 2013). ABCB1 substrates preferentially accumulate in cholesterol-rich regions of the membrane, and their pumping activity is enhanced in the presence of cholesterol (Subramanian et al. 2016). In chemoresistant colon cancer HT29 (HT29-dx) cells, the reduction of cholesterol synthesis is regulated by attenuated activity and expression of HMG-CoAR through induction of the E3 ligase Trc8, reducing cell viability (Gelsomino et al. 2013). This change is accompanied by decreased ABCB1 surface level in HT29-dx cells, which may exert a pro-immunogenic effect in response to chemotherapy through the restoration of extracellular HMGB1 (high mobility group box 1) expression, an index of necrosis and immunogenic death (Gelsomino et al. 2013). These data suggest that ABCB1 not only participates in cholesterol transport within the plasma membrane but also contributes to the development of chemoresistance in response to anticancer drugs.

#### ABCG1

ABCG1, also known as ABC transporter G1, participates in the process of reverse cholesterol transport by expelling excess cholesterol from cells into high-density lipoprotein particles (Kennedy et al. 2005). A deficiency of ABCG1 in mice significantly inhibits the subcutaneous growth of B16-melanoma and MB49-bladder carcinoma cells (Sag et al. 2015). In addition, the absence of ABCG1 in macrophages has been demonstrated to enhance their inflammatory characteristics and diminish the growth of subcutaneous tumors in mice following a Western-like diet (Sag et al. 2015). In *Abcg1−*/− mice, tumor growth is linked to a change in the macrophage phenotype within the tumor, transitioning from a tumor-promoting M2 to a M1 tumor-fighting profile (Sag et al. 2015). In lung cancer HKULC4 cells, the overexpression of ABCG1 not only stimulates cell proliferation but also increases the expression of anti-apoptotic proteins, namely BCL2 and MCL1 (Tian et al. 2017a, b). Enhanced cholesterol efflux promotes IL-4-mediated reprogramming, which includes the suppression of gene expression induced by IFNγ (Goossens et al. 2019). Genetic deletion of ABCG1 reverses the tumor-promoting functions of TAMs and leads to a reduction in tumor burden (Goossens et al. 2019). Various ABC transporters, including ABCG1, are considered as therapeutic targets for improving cancer treatment.

## Targeting lipid metabolism for cancer treatment

**Fig. 2:**
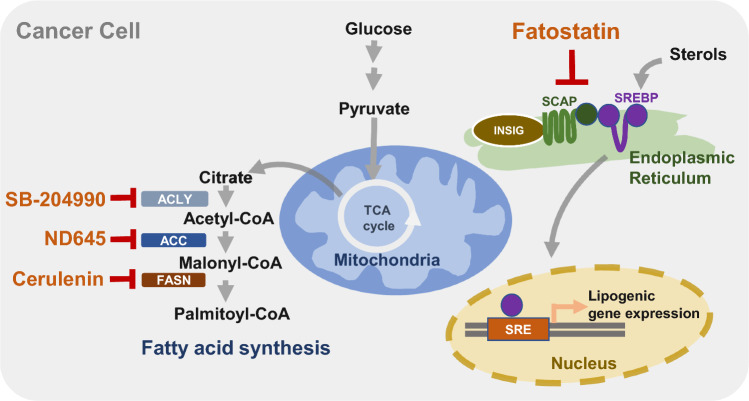
Modulation of lipid metabolism by small molecular inhibitors to treat cancer. Small molecules specifically inhibit their target enzymes involved in lipid metabolism through direct binding and/or suppression of activity (Fatostatin, SREBP inhibitor; Cerulenin, FASN inhibitor; ND-654, ACC inhibitor; SB-204990, ACLY inhibitor). As a consequence, altered lipid modulation remodels TME less favorable for cancer promotion and progression

**Fig. 3:**
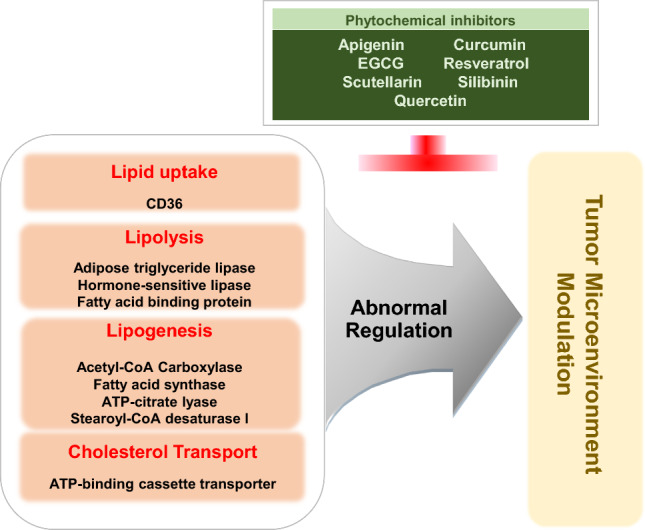
Potential multi-target effects of dietary phytochemicals on lipid metabolism in cancer. Unlike small molecular inhibitors, dietary phytochemicals simultaneously modulate multiple targets involved in lipid metabolism, rather than triggering specific single enzyme. This multi-target approach can influence various aspects of TME, while preventing development of resistance to a single pathway

In the preceding section, we explored the fundamental factors that underlie the reprogramming of lipid metabolism, which propels the progression of cancer. Furthermore, we assessed the interplay between cancer progression and altered lipid metabolism, encompassing crucial processes such as lipid uptake, lipolysis, and lipogenesis within the TME. With a focus on these factors responsible for lipid metabolic reprogramming, driving cancer progression, there is considerable potential in devising novel approaches for cancer treatment. Notably, clinical trials are currently evaluating the efficacy of fluvastatin, simvastatin, and denifanstat, which target lipid metabolisms such as HMG-CoAR or FASN in several carcinomas, including NSCLC, breast cancer, ovarian cancer, and prostate cancer (Han et al. 2011; Longo et al. 2020; Falchook et al. 2021). In addition, it is well documented that a variety of phytochemicals suppress cancer development and progression through modulating lipid metabolism. While small molecules trigger a specific target to regulate lipid metabolism (Fig. 2), phytochemicals often have the advantage of simultaneously affecting multiple targets (Fig. 3). In this context, we introduce a range of small molecular inhibitors and a variety of phytochemicals that target lipid metabolism, expanding beyond those previously mentioned.

### Small molecular inhibitors

#### Fatostatin

Fatostatin is a small molecule non-sterol diarylthiazole derivative that is a specific inhibitor of the SREBP cleavage-activating protein (SCAP) required for SREBP activation (Fig. 2, Table 1). It can bind to SCAP directly, inhibiting the activation of SREBP (Kamisuki et al. 2009). Targeting the SREBP-mediated lipogenic program by fatostatin led to a blockade of tumor growth as well as distant metastasis by decreasing the frequency of mitotic cancer cells in the *Pml*/*Pten* double-null mouse model of prostate cancer (Chen et al. 2018). Fatostatin decreases the expression of SREBP target genes such as *FASN*, *SCD-1*, *HMGCR*, *HMGCS1*, *MVK*, *MVD*, and *LDLR* in prostate cancer cells, followed by a reduction in intracellular fatty acid and total cholesterol levels (Li et al. 2014). Similar findings have been reported in human endometrial carcinoma cells, and an antitumor effect was also observed in a subcutaneous HEC-1A endometrial and androgen-insensitive C4-2B prostate xenograft model (Li et al. 2014; Yao et al. 2020). In addition, temozolomide-resistant glioblastoma multiforme U87 (U87R) cells exhibit a significant increase in cholesterol and fatty acid synthesis, alongside the reduction of lipid unsaturation, resulting in diminished membrane fluidity (Choo et al. 2023). Fatostatin shows more potent cell growth inhibitory effects in U87R cells compared to sensitive U87 cells (Choo et al. 2023). Moreover, fatostatin leads to a significant decrease in the level of the active form of SREBP1 in pancreatic cancer MIAPaCa-2 cells. This reduction is associated with cytotoxicity and reduction of proteins involved in lipid biosynthesis such as FASN, SCD-1, and HMG-CoAR (Siqingaowa et al. 2017). Similar outcomes have been observed in various cell lines such as glioma, prostate, and breast cancer, demonstrating the potential of fatostatin to attenuate tumor growth (Gholkar et al. 2016).

**Table 1:** Effects of small molecule inhibitors of lipid metabolism on TME

Categories	Small molecule inhibitors	Molecular targets	Chemical structure	Effects on TME	References
Non-sterol diarylthiazole derivative	Fatostatin	**•** Directly bind to SCAP **•** Inhibit SREBP activation		**•** Reduce tumor growth and metastasis in prostate cancer mouse model **•** Anti-cancer effects in human endometrial carcinoma and prostate cancer xenograft models **•** Inhibit tumor growth in glioma, prostate, and breast cancer cell lines	Chen et al. (2018) Yao et al. (2020) and Li et al. (2014) Gholkar et al. (2016)
Antifungal antibiotic	Cerulenin	**•** Directly bind to FASN		**•** Suppress proliferation of human prostate cancer cells **•** Reduce tumorsphere formation **•** Reduce invasiveness and stemness in patient-derived glioma stem cells **•** Inhibit migration and invasion of cervical cancer cells **•** Reduce lymph node metastasis in the metastatic mouse model of cervical cancer	Nishi et al. (2016) Brandi et al. (2017) Yasumoto et al. (2016) Du et al. (2022) Du et al. (2022)
Liver-specific ACC inhibitor	ND-654	**•** Bind to ACC **•** Inhibit ACC phosphorylation by AMPK		**•** Suppress proliferation of human hepatic carcinoma cells	Lally et al. (2019)
γ-Lactone prodrug	SB-204990	**•** Inhibit ACLY		**•** Disrupt the mitochondrial membrane potential and inhibit cell growth in various human cancer cell lines **•** Inhibit growth of tumor xenografts	Hatzivassiliou et al. (2005) Hatzivassiliou et al. (2005)

#### Cerulenin

Cerulenin covalently binds to the ketoacyl synthase domain of FASN (Fig. 2, Table 1) (Kuhajda et al. 2000) to significantly suppress the proliferation of human pancreatic cancer MiaPaCa-2 and AsPC-1 cells (Nishi et al. 2016). In addition, treatment of tumorsphere Panc1 cells with cerulenin leads to reduced cell viability and significant changes in cell morphology. There is also a reduction in spheroids, suggesting cytoskeletal reorganization (Brandi et al. 2017). Similar to the findings of these studies, treatment with cerulenin, an inhibitor of FASN, reduced the invasiveness of patient-derived glioma stem cells as well as the expression of stemness markers such as CD133 and Sox2. These findings imply that FASN-driven de novo lipogenesis plays a crucial role in maintaining cancer stemness (Yasumoto et al. 2016). Consistent with this, impeding the activity of FASN by cerulenin in SKBR3 results in high impairment of cellular proliferation (Stoiber et al. 2018). Recently, Du et al*.* demonstrated that cerulenin impairs the migration and invasion of cervical cancer, specifically HeLa and CaSki cells, through cholesterol reprogramming (Du et al. 2022). Moreover, cerulenin effectively reduced the lymph node volumes and the percentage of popliteal lymph nodes in a metastatic mouse model of cervical cancer (Du et al. 2022).

#### ND-654

ND-646 and ND-654 compounds bind to the arginine residue within the biotin carboxylase domain of the ACC protein (Fig. 2, Table 1), effectively inhibiting both its dimerization and enzymatic activity. While ND-646 is broadly distributed throughout the body, ND-654 is designed to facilitate increased uptake in the liver (Svensson et al. 2016; Lally et al. 2019). Treating hepatic cellular carcinoma (HCC) HepG2 cells with liver-specific ND-654 leads to a reduction in the level of ACC phosphorylation by AMPK, which is followed by a decrease in cell proliferation. Interestingly, HepG2 cells expressing a mutation in ACC serine phosphorylation sites exhibit enhanced lipogenesis and proliferation (Lally et al. 2019). These studies provide valuable insights into the significance of de novo lipogenesis and dysregulation of AMPK-mediated ACC phosphorylation in the development of HCC. Furthermore, the findings highlight the potential of ACC inhibitors as a targeted approach for cancer treatment.

#### SB-204990

SB-204990, a γ-lactone prodrug of the potent ACLY inhibitor SB-201076 (Fig. 2, Table 1), effectively suppresses the synthesis of cholesterol and fatty acid in both HepG2 cells and rats (Pearce et al. 1998). In various human cancer cell lines, SB-204990 disrupts the mitochondrial membrane potential, impedes cell cycle progression, and inhibits cell growth. These effects are closely linked to the impairment of glycolytic metabolism and glucose-dependent lipid synthesis (Hatzivassiliou et al. 2005). Notably, intraperitoneal administration of SB-204990 significantly inhibits tumor growth in nude mice with xenografts from mouse pancreatic ductal cell lines carrying oncogenic *K-ras*^G12D^ alleles (Hatzivassiliou et al. 2005). Furthermore, treatment with SB-204990 in K-Ras-driven cancer cells results in a significant loss of cell viability in the absence of serum and during glutamine deprivation (Hatzivassiliou et al. 2005; Hatipoglu et al. 2022; Sola-Garcia et al. 2023). From the studies, ACL is identified as a potential therapeutic target and provides the rationale for the development of ACL inhibitors for cancer treatment.

### Phytochemicals

#### Apigenin

Apigenin is a flavonoid abundant in parsley, onions, oranges, and chamomile and is effective in treating asthma, shingles, and cancer (Fig. 3, Table 2) (Su et al. 2020a, b). In combination with other chemotherapeutics, such as doxorubicin, apigenin can sensitize inhibitory effects on tumor growth and proliferation (Gao et al. 2013, 2018; Nozhat et al. 2021). In addition to anti-cancer effects, apigenin plays a role in lipid metabolism and has been reported to attenuate lipid accumulation in human hepatic cancer cell lines and adipocytes (Ono and Fujimori 2011; Lu et al. 2019; Hsu et al. 2021). In a high-fat diet (HFD)-induced obese mouse model, apigenin reduced body weight and visceral adipose tissue through downregulation of FASN, SCD1, or CD36, suggesting an anti-adipogenic effect (Su et al. 2020a, b; Wu et al. 2021). Moreover, apigenin can activate lipolysis. In human hepatocarcinoma HepG2 cells, apigenin-induced autophagy-dependent lipid degradation (Lu et al. 2020). Similarly, apigenin treatment upregulated the expression of genes responsible for lipolysis, including ATGL, HSL, FOXO1, and ACC in adipose tissues of HFD-induced obese mice (Table 2) (Sun and Qu 2019). These data suggest that apigenin can exert an anti-cancer effect by regulating lipid metabolism, although further investigation is required to confirm the phenomenon in vitro and in vivo.

**Table 2:** Effects of phytochemicals on lipid metabolism

Categories	Phytochemicals	Origins	Chemical structure	Effects on lipid metabolism	References
Flavonoid	Apigenin	Parsley, Onion, Chamomile		**•** Reduce lipid accumulation in cancer cells and adipocytes **•** Promote lipid degradation in HepG2 cells **•** Reduce visceral adipose tissues (↓FASN, SCD1, CD36) in HFD mice **•** Induce lipolysis-responsible genes (ATGL, HSL, FOXO1, and ACC) in HFD mice	Ono and Fujimori (2011), Lu et al. (2019), and Hsu et al. (2021) Lu et al. (2020) Su et al. (2020a, b), and Wu et al. (2021) Sun and Qu (2019)
Polyphenol	Curcumin	*Curcuma longa*		**•** Inhibit lipid accumulation and adipogenesis in adipocytes (↑ACC phosphorylation, fatty acid oxidation) **•** Reduce fatty acid uptake and biosynthesis (↓CD36, FASN) in NAFLD mice **•** Promote cholesterol efflux in macrophages	Ejaz et al. (2009) Yan et al. (2018) Lin et al. (2015), and Tan et al. (2021)
Polyphenolic flavonoid	EGCG	Green tea		**•** Inhibit lipid accumulation, dipogenesis, and cell proliferation in adipocytes **•** Promote lipolysis (↑HSL, autophagy) **•** Reduce white adipose tissue weight in HFD mice **•** Inhibit lipogeneic gene expression (ACC1, FASN, SCD1) **•** Induce lipolysis gene expression (HSL, ATGL) **•** Decrease fatty acid synthesis in cancer cells and xenograft tumors (↓FASN, ACC, ACLY) **•** Reduce triglyceride level and inhibits lipolysis in Huh7 cells **•** Promote fat clearance in cancer cells (↑autophagy)	Moon et al. (2007), and Kim and Sakamoto (2012) Lee et al. (2009), Zhou et al. (2014), and Kim et al. (2017) Choi et al. (2020) Li et al. (2018), Choi et al. (2020), and Lee et al. (2009) Khiewkamrop et al. (2022) Suihara et al. (2021) Zhou et al. (2014)
Polyphenol	Resveratrol	Grapes, Berries		**•** Inhibit adipogenesis (↓FASN, PPARγ) in adipocytes **•** Reduce lipid accumulation in HFD mice **•** Increase fatty acid oxidation (↓ACC activation) in mice **•** Promote cholesterol efflux in macrophages	Rayalam et al. (2008), Santos et al. (2014), and Chang et al. (2016) Kim et al. (2011), and Andrade et al. (2014) Gimeno-Mallench et al. (2019) Voloshyna et al. (2013), and Ye et al. (2019b)
Flavonoid	Scutellarin	Scutellaria baicalensis		**•** Inhibit adipogenesis in adipocytes **•** Inhibit hepatic lipid accumulation in HepG2 and HFD mice (↓CD36, FASN ACC)	Lu et al. (2013) Luan et al. (2020), and Zhang et al. (2022b)
Flavonoid	Silibinin	Milk thistle		**•** Inhibit lipid accumulation and proliferation in adipocytes (↓ FASN) **•** Reduce white adipose tissue weight and triglyceride accumulation in HFD mice **•** Inhibit lipid accumulation in HepG2 cells and tumor xenografts (↓ FASN, ACC)	Yang et al. (2021) Alsaggar et al. (2020) Deep et al. (2017)
Flavonoid	Quercetin	Red onion, Apple, Berries, Citrus fruits		**•** Inhibit differentiation and lipid accumulation in adipocytes **•** Reduces white adipose tissues and hepatic lipid accumulation in HFD mice (↓ FASN, CD36) **•** Inhibits fatty acid accumulation in HepG2 cells (↓ FASN)	Hong et al. (2021), and Seo et al. (2015) Jung et al. (2013) Li et al. (2013), and Zhao et al. (2014)

#### Curcumin

Curcumin is a well-known chemopreventive phytochemical extracted from the rhizome of *Curcuma longa* that is widely used as a spice in Asian countries (Table 2) (Tomeh et al. 2019). In addition to anti-inflammatory and anti-carcinogenic effects, curcumin is closely associated with lipid metabolism (Nosrati-Oskouie et al. 2021). Curcumin facilitates ACC phosphorylation and fatty acid oxidation in 3T3-L1 adipocytes, while inhibiting lipid accumulation and adipogenesis (Ejaz et al. 2009; Tian et al. 2017a, b; Wu et al. 2019). In HFD-induced obese mice, dietary curcumin reduced body weight gain, body fat, lipid levels, and hepatic lipid accumulation (Ejaz et al. 2009; Kobori et al. 2018). Yan et al. have demonstrated that oral administration of curcumin reduced CD36 and FASN expression in the liver of NAFLD mice (Table 2), implying its inhibitory effects on fatty acid uptake and biosynthesis (Yan et al. 2018). Moreover, curcumin promoted cholesterol efflux via ABCA1 in THP-1 macrophages to prevent atherosclerosis (Lin et al. 2015; Tan et al. 2021). In line with that, randomized controlled trial studies showed that curcumin supplementation improved lipid profiles in individuals at risk of cardiovascular diseases, polycystic ovary syndrome, and metabolic syndrome, further supporting the effects of curcumin on lipid metabolism (Tabrizi et al. 2018; Sohaei et al. 2019; Rafiee et al. 2021).

Curcumin functions in the lipid metabolism of various types of cancers, inducing apoptosis in human breast cancer MDA-MB-231 and liver cancer HepG2 cell lines, which is mediated by the downregulation of FASN (Fan et al. 2014, 2016). In addition to its inhibitory effect on lipogenesis, curcumin decreased cholesterol absorption into human colorectal cancer Caco-2 cells, suppressing cancer cell proliferation (Qin et al. 2023). Moreover, curcumin attenuated stem cell phenotypes of human breast cancer cells through the downregulation of fatty acid desaturases, such as SCD, FADS1, and FASD2 (Colacino et al. 2016). In tumor-bearing mice, curcumin combined with sorafenib enhanced serum lipid profiles and reduced FASN expression compared to the sorafenib-only-treated group (Man et al. 2020). Moreover, curcumin further inhibited tumor growth and EMT in sorafenib-treated mice, which suggests that curcumin sensitizes cells to the anti-cancer effects of sorafenib through alteration of lipid metabolism (Man et al. 2020). Taken together, these results suggest that curcumin exerts anti-tumorigenic effects by altering lipid metabolism (Fig. 3).

#### Epigallocatechin gallate (EGCG)

EGCG, a polyphenolic flavonoid, is a main bioactive ingredient in green tea that has been found to possess diverse biological functions and health benefits, particularly in cancers and metabolic syndrome (Legeay et al. 2015; Alam et al. 2022). EGCG has been reported to inhibit adipogenesis, lipid accumulation, and cell proliferation in 3T3-L1 adipocytes (Moon et al. 2007; Kim and Sakamoto 2012). In addition to the anti-lipogenic effect, EGCG promoted HSL-mediated lipolysis in 3T3-L1 cells (Lee et al. 2009). Moreover, EGCG led to autophagy-induced lipolysis in vivo and in vitro (Zhou et al. 2014; Kim et al. 2017). These lipid-modulating effects of EGCG have been demonstrated in diet-induced obese animal models as well. EGCG intake reduced body weight gain and white adipose tissue weight in HFD-induced obese mice (Choi et al. 2020). Moreover, EGCG significantly improved serum lipid profiles in HFD-fed mice and rats (Li et al. 2018; Li and Wu 2018). EGCG can stimulate lipid catabolism through inhibition of the expression of lipogenic genes such as ACC1, FASN, and SCD1 and through induction of lipolysis-involved genes, like HSL and ATGL (Table 2) (Lee et al. 2009; Li et al. 2018; Choi et al. 2020).

In cancer settings, EGCG has been reported to suppress tumorigenesis by regulating lipid metabolism. EGCG treatment resulted in increased apoptosis and decreased fatty acid synthesis through downregulation of FASN, ACC, and ACLY in human colorectal cancer cell lines as well as in a tumor xenograft mouse model (Khiewkamrop et al. 2022). Similarly, EGCG inhibited FASN and subsequent fatty acid synthesis, which mediated apoptosis in human prostate and liver cancer cells (Brusselmans et al. 2003; Khiewkamrop et al. 2022). Moreover, EGCG markedly reduced intracellular triglyceride levels and accelerated lipolysis in human hepatoma Huh7 cells (Suihara et al. 2021). In particular, EGCG can stimulate autophagy-dependent fat clearance in human liver cancer cells (Zhou et al. 2014). These data suggest a cross-link between the lipid-modulating and anti-carcinogenic effects of EGCG.

#### Resveratrol

Resveratrol is a potent anti-oxidative phytochemical mainly found in grapes and berries and can effectively prevent cardiovascular diseases, carcinogenesis, and metabolic syndrome (Table 2) (Ko et al. 2017; Hou et al. 2019). In particular, resveratrol activates SIRT1 but downregulates PPARγ, implying its role in lipid metabolism. In 3T3-L1 adipocytes, resveratrol suppresses adipogenesis through the downregulation of FASN and PPARγ and leads to apoptosis (Rayalam et al. 2008; Santos et al. 2014; Chang et al. 2016). In HFD-fed obese mice, resveratrol can reduce body weight and lipid accumulation, while enhancing lipid profiles, which seems to be mediated by the expression of key adipogenic genes, such as SREBP-1 and FASN (Kim et al. 2011; Andrade et al. 2014). Consistently, a meta-analysis showed that resveratrol intake can enhance lipid profiles in obese people (Zhou et al. 2022). Konings et al. have shown that resveratrol intake reduced abdominal subcutaneous adipocyte size in obese men (Konings et al. 2014). These data suggest that resveratrol possesses an anti-adipogenic effect in vitro and in vivo. Moreover, resveratrol affects lipolysis and cholesterol transport, facilitating basal and/or drug-induced triglyceride hydrolysis in fat cells (Szkudelska et al. 2009; Gomez-Zorita et al. 2013). Resveratrol upregulated ATGL expression at mRNA and protein levels in 3T3-L1 cells (Lasa et al. 2012). In old healthy mice, resveratrol inhibited the expression and activation of ACC1, resulting in increased fatty acid oxidation (Gimeno-Mallench et al. 2019). Of note, resveratrol accelerated ABCA1/ABCG1-mediated cholesterol efflux in human and murine-derived macrophages, implying its preventive role in atherosclerosis (Voloshyna et al. 2013; Ye et al. 2019a, b). Overall, resveratrol is involved in diverse processes of lipid metabolism, conferring protection against obesity and cardiovascular diseases.

Regarding its anti-carcinogenic and lipid-modulating effects, resveratrol has been thought to suppress tumor promotion and progression by altering lipid metabolism. Resveratrol significantly inhibited lipid synthesis, cell proliferation, and stemness by downregulating the expression of lipogenic genes, such as SREBP1 and its downstream ACLY1, ACC1, and FASN, in human breast and pancreatic cancer cells (Pandey et al. 2011; Khan et al. 2014; Zhou et al. 2019). Consistently, resveratrol inhibited adipocyte hypertrophy in the mammary fat pad of HFD-fed obese mice, leading to suppression of tumor growth (Rossi et al. 2018). In hepatoma-bearing rats, resveratrol intake reduced tumor size, tumor metastasis, and serum cholesterol level (Miura et al. 2003). Fukuda et al. have shown that resveratrol inhibited oral cancer cell growth by blocking epidermal FABP expression in vitro as well as in the xenograft model (Fukuda et al. 2022). Taken together, these findings indicate that resveratrol can inhibit lipogenesis and lipid uptake, leading to the suppression of tumor promotion and progression.

#### Scutellarin

Scutellarin is isolated from *Scutellaria baicalensis*, an herb widely used in Chinese medicine to treat inflammatory diseases and hyperlipidemia (Table 2) (Wang and Ma 2018). Scutellarin has been reported to exert chemopreventive and anti-cancer effects in various types of cancers (Li et al. 2010; Wang et al. 2010; Xu and Zhang 2013). In terms of lipid metabolism, scutellarin inhibited adipogenesis through PPARα in 3T3-L1 cells (Lu et al. 2013). Moreover, scutellarin attenuated hepatic lipid accumulation in both HepG2 cells and HFD-fed mice, which was mediated by the upregulation of CD36, FASN, and ACC (Luan et al. 2020; Zhang et al. 2022a, b). In line with that, scutellarin can protect against nonalcoholic fatty liver diseases and hyperlipidemia in animal models (Fan et al. 2017; Zhang et al. 2022a, b). Thus, scutellarin is a feasible candidate for suppressing tumor promotion and progression through the modulation of adipogenesis.

#### Silibinin

Silibinin is a flavonoid derived from the seeds of *Silybum marianum* (milk thistle) and is well-known for its hepatoprotective and chemopreventive activities (Table 2) (Abenavoli et al. 2010; Cheung et al. 2010). It has been reported that silibinin can improve lipid metabolism in obese mice, based on proteomics analysis (Wang et al. 2020a, b, c). In adipocytes, silibinin suppressed lipid accumulation and cell proliferation, possibly through the downregulation of FASN (Ka et al. 2009; Suh et al. 2015). In HFD-fed obese mice, silibinin intake reduced body weight gain, white adipose tissue weight, and adipocyte hypertrophy (Alsaggar et al. 2020). In the liver of HFD-fed mice, silibinin attenuated the expression of FASN and ACC and triglyceride accumulation, protecting against liver steatosis (Yang et al. 2021).

Silibinin can affect tumorigenesis through lipid modulation as well. Silibinin has been reported to attenuate adipogenesis in various types of cancers. In HepG2 cells, silibinin inhibited the expression of FASN and ACC and lipid deposition (Yang et al. 2021). In human endometrial and prostate cancer cell lines, silibinin effectively blocked the expression and/or activation of SREBP1, SCD-1, FASN, and ACLY, resulting in suppression of lipid accumulation and cancer cell proliferation (Nambiar et al. 2014; Shi et al. 2019). Interestingly, silibinin suppressed expression levels of genes responsible for lipogenesis and cell invasion in HepG2 cells exposed to sera from obese males, suggesting silibinin as a potential chemotherapeutic in obesity-induced cancer (Miethe et al. 2017). Similarly, silibinin inhibited hypoxia-induced lipogenesis and angiogenesis under hypoxic conditions in human prostate cancer cells (Deep et al. 2017). Furthermore, silibinin administration dramatically decreased tumor growth, intratumoral lipid accumulation, and expression levels of FASN and ACC in the tumor xenograft mouse model (Deep et al. 2017). Thus, silibinin may be useful for the treatment of cancer patients with highly lipogenic phenotypes.

#### Quercetin

Quercetin is a dietary flavonoid abundant in red onions, apples, berries, and citrus fruits that exhibits antioxidant, anti-obesity, and anti-cancer effects (Table 2) (Shabir et al. 2022). In adipocytes, quercetin inhibited differentiation and fat accumulation in 3T3-L1 cells (Hong et al. 2021). Quercetin provoked upregulation of ATGL and HSL expression and downregulation of FASN and LPL expression in OP9 cells that differentiate into adipocytes (Seo et al. 2015). In HFD-induced obese mice, dietary quercetin significantly reduced body weight gain, white adipose tissues, and hepatic lipid accumulation (Jung et al. 2013). Moreover, quercetin attenuated expression levels of CD36 and FASN in the liver of these obese mice (Jung et al. 2013). Thus, quercetin exerts its anti-obesity effect by preventing adipogenesis and promoting lipolysis.

In HepG2 liver cancer cells, quercetin downregulated FASN expression and accumulation of intracellular fatty acids, resulting in increased apoptosis (Li et al. 2013; Zhao et al. 2014). Similarly, quercetin blocked FASN expression and cell proliferation in human nasopharyngeal carcinoma cell lines (Daker et al. 2013). Quercetin intake suppressed tumor size and lipid accumulation in tumor-bearing mice, suggesting a correlation between the anti-cancer and lipid-modulating effects of quercetin (Ruidas et al. 2022). In Fig. 3, an illustration is presented depicting proteins related to lipid metabolism that can be influenced by phytochemicals.

## Concluding remarks and perspectives

Metabolic reprogramming within the TME has been recognized as a crucial hallmark of cancer (Pavlova and Thompson 2016). Tumors require a huge supply of energy to expedite promotion and progression. To date, investigations into metabolic alteration in cancer have focused on glucose utilization, referred to as the Warburg effect. Lipid metabolism is emerging as another key player in TME-related metabolic reprogramming (Snaebjornsson et al. 2020). TME represents altered lipid metabolic pathways, which are closely associated with the activation of oncogenic signals (Fernandez et al. 2020). Thus, reprogrammed lipid-modulating genes in TME may be novel targets for the prevention and treatment of cancers. Targeting lipid metabolic reprogramming factors that promote cancer progression would support new strategies for cancer treatment. As numerous phytochemicals exert anti-obesity and anti-cancer effects concomitantly, it would be worth repurposing lipid-modulating drugs and/or phytochemicals as chemotherapeutics. Therefore, further investigations are needed to fully understand the underlying mechanisms and the correlation between altered lipid metabolism and tumorigenesis.
